# Psychological Mechanisms of Caregiver Involvement in Caregiving for Individuals with Alzheimer’s Disease: Analysis of the Moderated Mediation Model

**DOI:** 10.3390/jcm14145134

**Published:** 2025-07-19

**Authors:** Anna Sołtys, Marcin Wnuk

**Affiliations:** 1 Faculty of Social Sciences, Institute of Psychology, University of Szczecin, 70-453 Szczecin, Poland; 2 Faculty of Psychology and Cognitive Sciences, Adam Mickiewicz University, 61-712 Poznan, Poland; marwnu@amu.edu.pl

**Keywords:** caregiver stress, sense of coherence, involvement in caregiving, Alzheimer’s disease

## Abstract

Providing long-term care for a person with Alzheimer’s disease is associated with chronic stress and emotional overload. One of the key predictors of emotional burden is the amount of time devoted to caregiving, which intensifies the experienced stress. Additional risk factors include the stage of the illness, difficulties in the care recipient’s activities of daily living, the caregiver’s neglect of their own needs, and challenging behaviours exhibited by the person receiving care. Therefore, it is essential to identify the psychological protective resources of caregivers that can buffer the impact of stress. **Background/Objectives**: The objective of the study was to explore the psychological mechanisms underlying the involvement of caregivers supporting individuals with Alzheimer’s disease. A moderated mediation model was employed, in which stress indirectly affects caregiver involvement through a sense of coherence, and the strength of this relationship is moderated by the amount of time devoted to caregiving. **Methods**: The bootstrapping method was applied using 5000 resamples within a 95% bias-corrected confidence interval. The analysis accounted for variables such as stress levels, sense of coherence, involvement in caregiving, duration of care, education, gender, age, and stage of the illness. **Results**: The sense of coherence mediated the relationship between stress and involvement in caring (B = 0.0063, SE = 0.0031, 95% CI [0.0012, 0.0135]), and this indirect effect was contingent upon the amount of time devoted to helping. The relationship between sense of coherence and involvement in caring was significant at the mean level (B = 0.005, SE = 0.002, 95% CI [0.0004, 0.0101]) and became stronger at high levels of time devoted to caring (+1 SD; B = 0.009, SE = 0.003, 95% CI [0.0030, 0.0148]). These results indicate that the positive association between sense of coherence and caregiver involvement increases with the amount of time spent caring. **Conclusions**: The results highlight the importance of strengthening caregivers’ resilience resources—such as a sense of coherence—in preventing overload. The model may serve as a foundation for developing interventions aimed at supporting caregivers’ mental health.

## 1. Introduction

Alzheimer’s disease is an irreversible neurodegenerative condition that leads to a gradual loss of cognitive and functional abilities, accompanied by a range of neuropsychiatric symptoms, all of which significantly impair the care recipient’s quality of life [1]. As the disease progresses, individuals experience memory loss, mood swings, aggression, self-destructive behaviour, and difficulties in decision-making, resulting in a continual decline in independence [2,3,4]. Providing long-term care for a person with Alzheimer’s disease—which typically lasts between four and eight years—places a chronic emotional and physical burden on the caregiver. This can lead to the development of ‘caregiver stress syndrome’, characterised by exhaustion, increasing feelings of isolation, and various health problems [5,6,7,8], including reduced immunity, cardiovascular disease, sleep disturbances, heightened irritability [8,9], depression, anxiety disorders [10], and an elevated risk of premature death [11].

Numerous studies indicate that caregivers experience higher levels of stress than the general population. This increase is attributed to two main factors. The first is the growing amount of time devoted to caregiving, particularly as the care recipient’s cognitive impairments, behavioural disturbances, and affective or psychotic symptoms progress [12]. Caregivers who had the opportunity to rest or delegate their responsibilities for a few hours each day reported lower levels of stress compared to those engaged in continuous caregiving [13]. The daily amount of time devoted to caregiving is one of the most objective indicators of caregiver burden and is also a key factor that increases the risk of developing depressive symptoms [14,15,16,17]. Prolonged caregiving heightens experienced stress, which, over time, adversely affects the caregiver’s physical health and often leads to a range of somatic symptoms [18]. The second factor concerns the relationship with the care recipient and involves a perceived loss of control over the situation, interpersonal conflicts, lack of support, and financial, emotional, and physical strain [12]. The multidimensional burden placed on caregivers arises from the need to organise the care recipient’s daily routine, manage multiple roles simultaneously, and cope with the difficulty of accepting a new reality. Sacrificing personal needs in order to provide care can lead to internal conflict and ambivalent emotions [19]. The care recipient’s escalating symptoms of aggression and agitation often necessitate constant supervision, which significantly increases the demands placed on a caregiver and intensifies their overall burden [20]. The overload experienced by caregivers affects both their physical and emotional well-being. The physical dimension of this burden is associated with excessive responsibilities, lack of quality sleep and rest, as well as psychosomatic symptoms such as headaches, anxiety, and loss of appetite. The emotional dimension involves increasing levels of anxiety, frustration, helplessness, and a perceived lack of control. Long-term psychological strain is often linked to a sense of moral obligation towards the care recipient, which may lead to emotional burnout. In extreme cases, this can result in depersonalisation—a psychological distancing from the situation—which may manifest as aversion towards the care recipient, neglect of caregiving duties, and a loss of responsibility for assigned tasks [21,22].

The primary factors determining the level of burden placed on caregivers stem directly from the progression of the disease. This burden is also influenced by sociodemographic variables and caregiving-related aspects, such as the amount of time devoted to caregiving, the nature of the relationship between the caregiver and the care recipient, the caregiver’s gender, and the complexity of daily tasks [23,24]. Numerous studies have shown a strong association between caregiver burden and factors such as the age of the care recipient, the stage of illness [25], the number of hours spent caregiving—particularly during the night—and the family’s financial situation. A low income and high medical expenses further contribute to increased stress and overload [26]. Previous studies have not included the duration of caregiving as a moderator in mediation analyses; however, it has been shown to be one of the most important predictors of caregiver burden. This justifies examining its moderating role in the relationship between central resilience resources and caregiver burden. With greater amounts of time spent on caregiving, sense of coherence may be either weakened or strengthened, depending on the stress experienced by the caregiver. Demonstrating the potential role of caregiving time as a moderator may provide a better understanding of the conditions under which sense of coherence can protect caregivers from the negative effects of stress.

In the context of research on the psychosocial functioning of caregivers, it is essential to consider the issue of stress and its impact on health, particularly in relations to the perceived caregiving burden [27,28]. Individuals with a low sense of coherence and unstable personality traits are more vulnerable to stress; therefore, strengthening their resilience mechanisms is crucial for maintaining psychological balance [29]. In the literature, the term ‘caregiver stress’ refers to the burden associated with providing long-term care to a person with a chronic illness. Continuous exposure to mental and physical strain is one of the key factors disrupting the body’s homeostasis [30]. The caregiver stress model enables the identification of factors influencing the level of burden experienced by caregivers. Stress associated with this role arises from the combination of two main groups of stressors. The primary stressor is the illness itself, which, over time, increases the care recipient’s dependence and requires the caregiver to make significant decisions. Secondary stressors arise from the prolonged performance of the caregiving role and its associated consequences. The degree of stress experienced depends on the relationship between the demands placed on the caregiver and their individual capacity to cope with these challenges [31].

This concept aligns with the contemporary understanding of stress, which is perceived as a state of internal imbalance triggered by an actual or perceived discrepancy between environmental demands and individual’s ability to cope with them [32,33]. Restoring emotional stability requires actions aimed at alleviating the effects of stress. A crucial part of this process involves enhancing one’s sense of control over the situation, seeking information about effective coping strategies, reducing tension, maintaining positive self-esteem, limiting negative environmental influences, and re-evaluating perceived threats in order to strengthen psychological resilience to future challenges [34]. According to Lazarus and Folkman’s theory, a caregiver may perceive their situation as a loss or threat if the caregiving role restricts access to important values such as health, family, social relationships, or work. However, if personal goals remain attainable, the situation may instead be interpreted as a challenge [35]. Hobfoll, in contrast, highlights the importance of personal resources, which are often significantly depleted in caregivers due to the prolonged burden of care [32]. The general level of perceived stress measured in our study reflects only the intensity of experienced burden and does not in itself determine whether caregiving involvement will take an adaptive or maladaptive form. The ultimate nature of this involvement—whether it leads to beneficial or detrimental outcomes—may also depend on the caregiver’s available regulatory resources and the coping strategies they employ.

Caring for a person with Alzheimer’s disease involves chronic stress, which often leads to emotional exhaustion and a decline in the caregiver’s mental well-being [36]. Feelings of burnout and chronic fatigue among caregivers are not directly determined by the stage of the illness or the level of the care recipient’s disability, but rather result from a more complex interplay of factors. Research has identified specific patterns, including significantly higher levels of exhaustion among those caring for individuals with severe speech impairments [37]. A meta-analysis has shown that the decline in caregivers’ mental and physical well-being is reflected in elevated stress levels and an increased prevalence of depressive symptoms [38]. It has also been observed that risk of anxiety and depressive disorders is higher among caregivers who live with the care recipient, women, individuals in poor health, those experiencing strained relationships with the person they care for, and caregivers of individuals with more severe disabilities [39]. Chronic stress leads to disruptions in the immune and cardiovascular systems, primarily due to excessive activation of the nervous system, increased physiological reactivity, and elevated levels of epinephrine [40].

In situations that require coping with stress, a sense of coherence plays a crucial role by regulating coping strategies and mobilising internal resources [41]. As a central life orientation, a sense of coherence enables individuals to perceive reality as predictable and comprehensible, while also strengthening their sense of agency and capacity to overcome difficulties. It comprises three key components: comprehensibility, which refers to the cognitive appraisal of a situation and the belief that it is structured and manageable; manageability, which reinforces the belief that one possesses the necessary resources to cope with challenges, thereby enhancing a sense of security; and meaningfulness, which allows individuals to assign value and purpose to life events, motivating them to engage with and overcome adversity [42]. A sense of coherence enables individuals to manage their resources more effectively by directing them towards areas where they can realistically influence outcomes, while limiting energy expenditure on activities that are unlikely to produce desired results or may cause harm [43]. Life experiences play a role in shaping and modifying one’s sense of coherence. The most significant changes typically occur before the age of thirty years; however, exposure to new and enduring life situations can also influence it in later years. A decline in the sense of coherence is more likely among individuals exposed to sudden, unpredictable events, excessive stress, both overload and understimulation, as well as those with limited autonomy or decision-making power [44].

The research indicates that a sense of coherence plays a vital role in enabling caregivers to cope with the stress associated with their caregiving responsibilities. Chronic tension leads to the depletion of psychological resources, including a diminished sense of coherence, which undermines the ability to cope effectively with challenges. Caregivers who possess sufficient knowledge about the illness, understand the care recipient’s behaviour, find deeper meaning in their caregiving role, and recognise the value of their actions tend to demonstrate more effective coping strategies and are more likely to attend to their own needs. The sense of coherence model developed in relation to carers of people with dementia, including those caring for individuals with Alzheimer’s disease, suggests that, as the illness progresses, a gradual decline in coherence levels may occur [45]. Many researchers emphasise that a strong sense of coherence among caregivers significantly reduces the risk of mental health disorders, including depression [46], while also alleviating the subjective burden of care [47,48,49]. It promotes a more effective adaptation to caregiving demands, supports the attribution of meaning to personal experiences [50], and encourages the use of oriented strategies focused on seeking support and maintaining a future-oriented perspective [51].

In the literature on psychological resilience among caregivers, constructs such as self-efficacy, coping flexibility, and sense of coherence play a significant role. Generalised self-efficacy, according to Bandura’s theory [52], is considered a personality trait related to the belief in one’s own competence and ability to manage difficult situations, which is particularly relevant in the context of specific caregiving tasks. In contrast, coping flexibility [53] is associated with the appraisal of available coping strategies and the ability to adaptively switch strategies depending on the stressful situation, emphasising the importance of behavioural diversity and appropriateness. Sense of coherence, however, encompasses not only the ability to change strategies but also the perceived meaningfulness of the situation. According to Antonovsky [42], sense of coherence is a construct that integrates cognitive, behavioural (related to the instrumental function of coping), and emotional–motivational aspects. Unlike self-efficacy and coping flexibility, sense of coherence refers to a general life orientation and the way stress is interpreted as a manageable challenge, which makes it particularly useful as a model for studies of caregivers of people with Alzheimer’s disease, who experience chronic and often unpredictable burden. Therefore, in the present study, sense of coherence is adopted as the overarching theoretical framework, enabling a comprehensive understanding of caregiver’s resilience resources and informing the design of interventions to support their long-term wellbeing.

### Conceptual Model

**Figure 1:**
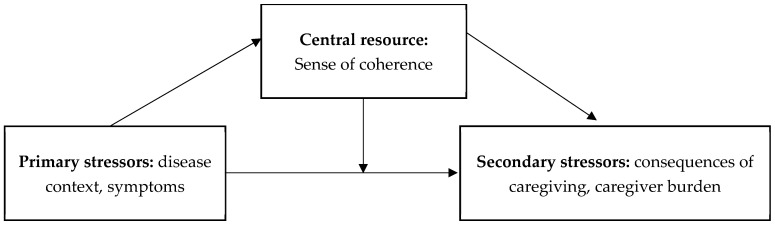
Conceptual model integrating Antonovsky’s salutogenic theory and Pearlin’s stress process model.

The theoretical model presented is grounded in the assumptions of Aaron Antonovsky’s salutogenesis theory [42] and the caregiver stress model [54]. It posits that the stress associated with the caregiving role influences the level of caregiver involvement through the mediating role of a sense of coherence. Additionally, the model incorporates the moderating effect of the amount of time devoted to caregiving. According to the caregiver stress model, prolonged exposure to stressors gradually increases the burden experienced by the caregiver [40]. It is assumed that a greater time commitment to caregiving amplifies the negative impact of a low sense of coherence on caregiver involvement, thereby increasing the risk of overload (Figure 1).

Pearlin’s caregiver stress model [54] distinguishes between primary stressors, which arise directly from the illness situation (such as the care recipient’s level of dependency and behavioural, emotional, or cognitive symptoms), and secondary stressors, which refer to the consequences of providing care (such as family conflicts, difficulties in balancing caregiving with professional roles, and caregiver burden). A high sense of coherence, as a central resilience resource in Antonovsky’s concept [42], plays an important role in reducing the negative impact of these stressors on caregivers’ functioning. It operates by enabling caregivers to perceive stressors as more comprehensible, to find meaning in their caregiving efforts, and to maintain confidence in their ability to cope with the caregiving role.

In addition, to more accurately reflect the realities of caregiving, the model included control variables such as the caregiver’s education and age, duration of care, and the stage of the illness. These variables may influence both stress levels and the caregiver’s capacity to cope with caregiving demands [17], and thus controlling for them allows for a more precise examination of the proposed relationships.

In summary, the model assumes a moderated mediation: stress influences involvement in caregiving through a sense of coherence, and the strength of this indirect effect is moderated by the amount of time devoted to caregiving.

Based on the proposed theoretical model, the aim of the study was to empirically examine the psychological mechanisms through which stress levels influence involvement in caregiving among caregivers of individuals with Alzheimer’s disease. Specifically, the study sought to determine the role of a sense of coherence as a mediator in this relationship, and the role of time devoted to caregiving as a moderator of the relationship between sense of coherence and caregiving involvement. The analysis also accounted for selected control variables, including age, caregiver’s level of education, duration of care, and stage of illness.

In light of the above, the following research hypotheses were formulated:

**H1:**  *The stress levels of caregivers of people with Alzheimer’s disease will be negatively associated with their sense of coherence; higher stress levels will be associated with lower levels of coherence.*

**H2:**  *The stress levels of those caregiving for people with Alzheimer’s disease will be positively associated with their involvement in caregiving; higher stress levels will correspond to greater involvement in caregiving.*

**H3:**  *A sense of coherence will be negatively associated with involvement in caregiving; a stronger sense of coherence will correspond to a lower level of involvement in caregiving.*

**H4:**  *A sense of coherence will mediate the relationship between stress levels and involvement in caregiving; higher stress levels will be indirectly associated with greater involvement in caregiving through a reduced sense of coherence.*

**H5:**  *The time devoted to caregiving will moderate the relationship between a sense of coherence and involvement in caregiving; with increased time devoted to caregiving, the negative relationship between a sense of coherence and involvement in caregiving will be stronger.*

**H6:**  *The indirect effect of stress on involvement in caregiving through a sense of coherence will be moderated by the time devoted to caregiving; the indirect effect will be stronger among caregivers who devote more time to caregiving.*

With regard to the control variables, an additional hypothesis was formulated:

**H7:**  *Age, education, duration of care, and stage of illness will be included as control variables due to their possible impact on a sense of coherence and involvement in caregiving, without formulating specific directional hypotheses.*

## 2. Materials and Methods

### 2.1. Subjects

The study sample comprised 100 caregivers of individuals with Alzheimer’s disease. The group consisted of 78% women and 22% men. The participants’ ages ranged from 19 to 90 years (M = 55.84). In terms of education, 47% had completed higher education, 44% secondary education, and 9% vocational training. The majority of caregivers (72%) had partners, 12% were single, 10% divorced, and 6% widowed. The duration of caregiving ranged from 1 to 19 years, with an average of 5.18 years (SD = 4.25). Within the group, 69% had been providing care for 1 to 5 years, 20% for 6 to 10 years, and 11% for 11 to 20 years. The number of hours devoted to caregiving also varied; 58% of caregivers provided care for more than 32 h per week, 28% for 17 and 32 h, and 14% for 8 and 16 h. The majority of care recipients (64%) were in stage II of dementia, 19% in stage III, and 17% in stage I.

Participation in the study was entirely voluntary. Potential participants were identified through medical records confirming their status as the primary caregiver of a person diagnosed with Alzheimer’s disease. A total of 140 caregivers were invited to take part in the study, of whom 100 ultimately participated. Each caregiver received written information outlining the purpose of the study and was also verbally briefed by a physician. Upon providing informed consent, participants completed a set of standardised questionnaires, either in the presence of the researcher or independently, depending on availability and individual circumstances. The identity of the care recipient and all personal data were treated as confidential and used solely for research purposes. The data collection process lasted approximately 10 months and was conducted both in care facilities and in home settings, following prior arrangements with the caregivers. The inclusion criteria for the study were: being the primary caregiver of a person of Alzheimer’s disease, aged over 18 years, providing care for a minimum of eight hours per week, having been in the caregiving role for at least one year, and providing informed consent to participate in the study. The exclusion criteria were being under 18 years of age, caring for a person with a type of dementia other than Alzheimer’s disease, circumstances involving the death of the care recipient, and caregivers whose health condition prevented them from completing the questionnaires independently.

The study employed a quantitative methodology with a single point of data collection. The data gathered included psychological variables (stress level, a sense of coherence, and level of involvement in caregiving), the number of hours per week devoted to caregiving, sociodemographic information (age, gender, education), duration of care, and the stage of illness experienced by the care recipient.

### 2.2. Investigation and Measurements

To collect data on the research variables, an original sociodemographic questionnaire and a set of standardised psychometric instruments were used.

#### 2.2.1. Sociodemographic Survey

For the purposes of the study, an original sociodemographic survey was developed to collect basic information characterising the respondents. The questionnaire included items on age, gender, level of education, duration of care, stage of illness, and the number of hours per week devoted to caregiving. The use of closed-ended questions facilitated the systematic analysis of quantitative data. The information obtained was used to establish a demographic profile of the participants and to examine the relationships between sociodemographic variables and the results obtained in subsequent parts of the study.

#### 2.2.2. Perceived Stress Questionnaire

The Perceived Stress Questionnaire (PSQ) consists of 27 items designed to assess the overall level of stress as well as its three core areas: emotional tension, external stress, and intrapsychic stress. The PSQ scales measure such aspects as anxiety, difficulty relaxing, chronic fatigue, frustration resulting from a sense of lack of control over the situation, emotional exhaustion, an impression that others are taking advantage of them, a reduced sense of meaning in life, helplessness, loneliness, and a lack of self-confidence. In validation studies, the Cronbach’s alpha for the entire scale ranged from 0.70 to 0.81. In our study, the Cronbach’s alpha was 0.84. Construct validity was confirmed by positive correlations with somatic and psychological symptoms and negative correlations with sense of coherence and psychological hardiness, ranging from r = −0.40 to −0.60. A factor analysis of the Polish adaptation confirmed its two-factor structure. Polish studies also demonstrated a high test-retest reliability over a period of 3–4 weeks (r = 0.72–0.81) [55].

#### 2.2.3. Orientation to Life Questionnaire

The Orientation to Life Questionnaire (SOC-29) consists of 29 items that measure a general sense of coherence and its three components: comprehensibility, manageability, and meaningfulness. The tool assesses the extent to which the study subject perceives the surrounding world as structured, predictable, and understandable; to what extent they believe in having adequate resources to cope with challenges; and how much meaning and value they attribute to their efforts in everyday life. This tool exhibits high reliability; the Cronbach’s alpha for the entire scale was 0.92, with values ranging from 0.68 to 0.78 for the subscales. In our study, the Cronbach’s alpha was 0.91. The adaptation confirmed both construct and factorial validity. Test–retest reliability over a six-month period was also demonstrated, with correlation coefficients ranging from r = 0.69 to r = 0.78 [42].

#### 2.2.4. Involvement Engagement Questionnaire

The Involvement Evaluation Questionnaire (IEQ) is used to assess the level of involvement of caregivers in caregiving for individuals with dementia in general. However, in the context of our study, it was used specifically in relation to carers of people with Alzheimer-type dementia. It consists of 27 items reflecting the experiences of caregivers. Two scales of the questionnaire—interpersonal tension and worrying—refer to the subjective sense of burden. Interpersonal tension assesses the relationship between the caregiver and the care recipient, the impact of the care recipient on the caregiver’s sleep, the sense of threat, and concerns about the future. Worrying refers to concerns about the safety, health, and future of the care recipient, along with the associated psychological burden. The other two scales—supervision and encouragement—assess the objective sense of burden. The supervision scale concerns actions aimed at controlling the care recipient’s behaviour, while the encouragement scale motivates the care recipient to be independent and the caregiver to take over the care recipient’s responsibilities. In Polish studies, the tool demonstrated high internal consistency, with the Cronbach’s alpha being equal to 0.90 for the entire scale and between 0.68 and 0.87 for the four subscales. Factor analyses confirmed a four-domain structure consistent with the original version, and construct validity was supported by significant correlations with other measures of caregiver burden. In our study, the Cronbach’s alpha was 0.92. In the current sample, the internal consistency coefficients (Cronbach’s alpha) for the subscales were as follows: Urging = 0.89, Supervision = 0.78, Tension = 0.88, and Worrying = 0.89, indicating good measurement reliability. The reliability of the European version of the IEQ is α = 0.7 [56].

### 2.3. Conceptual Model and Statistical Analysis

**Figure 2:**
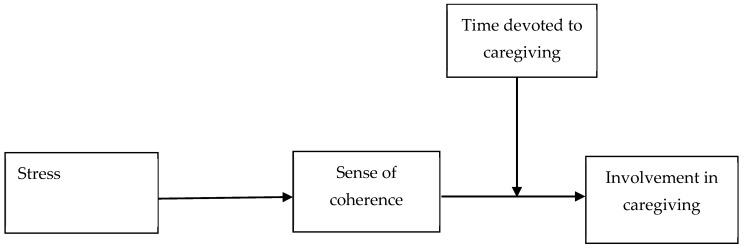
Conceptual model.

The theoretical model shown in Figure 2 reflects moderated mediation with stress as an independent variable, a sense of coherence as a mediator, involvement in caregiving as a dependent variable, and time devoted to caregiving as a moderator in the relationship between a sense of coherence and involvement in caregiving. The following controlled variables were used: education, sex, age, duration of caregiving, and stage of illness.

For the statistical analyses, IBM SPSS AMOS version 26.0 was used. Model number 14 of the PROCESS template for SPSS, authored by Hayes [57], was applied. The bootstrapping method was used based on 5000 resamples within a 95% bias-corrected confidence interval.

To assess if the sample size was large enough, a power analysis was applied, using G*Power 3.1.9.7. with linear multiple regression, a fixed model. A medium effect size of 0.15 with an alpha of 0.05, a power of 0.95, and 7 predictors was chosen. The results confirmed that 74 participants were the required sample size, which was 74% of the research sample.

When a moderation effect was found, the sample was divided into three groups, with analyses of moderation with probe interactions at −1 SD, mean, and +1 SD, reflecting ‘low’, ‘medium’, and ‘high’ values of that variable. Additionally, the Johnson–Neyman output was used [57] to examine the relationship between the independent variable and the dependent variable for regions of significance across levels of the moderator variable. The variance inflation factor (VIF) was employed to verify potential multicollinearity problems [58].

## 3. Results

**Figure 3:**
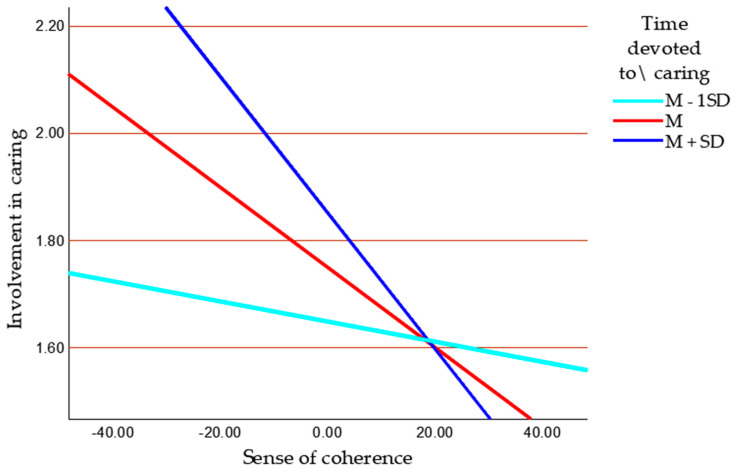
Moderating effect of time devoted to caregiving on the association between a sense of coherence and involvement in caregiving (*n* = 100). M—mean, SD—standard deviation.

Descriptive statistics are presented in Table 1. The VIF results were below the threshold of 5, which means that the data were not contaminated by multicollinearity. As shown in the results in Table 2, stress was negatively related to the sense of coherence and education and positively related to involvement in caregiving. The sense of coherence positively correlated with education, was negatively related to involvement in caregiving, and was unrelated to time devoted to caregiving. The association between time devoted to caregiving and involvement in caregiving was positive. A statistically significant negative direct effect of stress on the sense of coherence was observed, but not on involvement in caregiving, and the positive direct effect of time devoted to caregiving on involvement in caregiving was found (see Table 3). The negative direct effect of a sense of coherence on involvement in caregiving was observed. Among controlled variables, only the stage of illness was a positive predictor of involvement in caregiving. In the next stage of analysis, the interactive effect of a sense of coherence and time devoted to caregiving on involvement in caregiving was computed to determine if these variables interactively explain involvement in caregiving. An interactive effect of a sense of coherence and time devoted to involvement in caregiving was found (see Table 4) and explained an additional 3.35% of the variance of involvement in caregiving. The time devoted to caregiving strengthened the negative relationship between a sense of coherence and involvement in caregiving. This moderating effect is presented in Figure 3. In a group of caregivers who devoted less than the average time to caregiving, the relationship between a sense of coherence and involvement in caregiving was irrelevant (95% CI [−0.008, 0.006]; *p* = 0.832; b = −0.001) in comparison to statistically significant effects in caregivers who spent the average time for caregiving and more than average ((95% CI [−0.012, −0.001]; *p* = 0.032; b = −0.006) and (95% CI [−0.018, −0.003]; *p* = 0.004; b = −0.011), respectively).

A moderated mediation index (0.0063., SE = 0.0031. 95% CI [0.0012, 0.0135]) confirmed that a sense of coherence mediated the relationship between stress and involvement in caregiving, and this indirect relationship is moderated by the time devoted to caregiving. The moderating effects of stress on involvement in caregiving for different values of time devoted to caregiving, mediated by a sense of coherence, are presented in Table 5. The more time caregivers devote to caregiving, the stronger the positive indirect effect of stress on involvement in caregiving mediated by a sense of coherence.

## 4. Discussion

This study aimed to explore the mechanisms underlying the influence of stress on involvement in caregiving, with particular emphasis on the role of a sense of coherence and the amount of time devoted to caregiving. The results confirm all the research hypotheses, indicating consistency between the theoretical model and the empirical data obtained.

As expected, stress was significantly and negatively associated with a sense of coherence (H1), which aligns with previous research suggesting that higher levels of stress may impair an individual’s ability to perceive the world as coherent, comprehensible, and predictable. Caregivers with a strong sense of coherence are more likely to interpret their role as structured, meaningful, and manageable, which in turn contributes to lower levels of stress in everyday functioning. Thus, a sense of coherence may be regarded as a key psychological resource that protects caregivers and supports their ability to cope with long-term challenges [29,59,60].

Stress was also positively associated with involvement in caregiving (H2), suggesting that, under high-stress conditions, caregivers may exhibit increased involvement, potentially as a means of coping with a difficult situation or fulfilling a perceived sense of duty. Previous studies have shown that behaviours displayed by care recipients that deviate from social norms—such as aggression, irrationality, and unpredictability—combined with communication difficulties, significantly contribute to an increased subjective burden experienced by caregivers. This, in turn, leads to heightened anxiety and elevated stress levels. Furthermore, chronic role overload, insufficient personal time, and persistent fatigue contribute to a growing sense of anxiety and insecurity, which may ultimately hinder the effectiveness of caregiving [36,38,61,62,63]. Although it was assumed that higher levels of stress lead to changes in sense of coherence and involvement in caregiving, reverse causality is also possible, whereby greater involvement in caregiving may itself increase perceived stress levels. Caregivers who devote more time and energy to caregiving may experience a greater psychological burden associated with their caregiving responsibilities. Longitudinal and experimental studies are needed to determine the directionality of these relationships and to better understand the underlying mechanisms. The findings suggesting that experiencing stress may lead to increased involvement in caregiving can be interpreted in light of Lazarus and Folkman’s transactional model of stress [35]. This theory posits that stress arises when the demands of a situation exceed an individual’s coping resources, resulting in a state of imbalance. Consequently, the individual engages in efforts to mitigate the impact of the stressor and restore internal equilibrium. In the context of caregiving, elevated stress may prompt greater involvement as a compensatory response and an attempt to reduce the perceived threat associated with the care recipient’s deteriorating condition. Stress may, therefore, motivate caregivers to intensify their efforts, sometimes at the expense of their own health and well-being.

It was also observed that a stronger sense of coherence was associated with a lower level of involvement in caregiving (H3). This suggests that caregivers who have a better understanding and interpretation of the situation, and greater confidence in their ability to cope, may be more effective in distancing themselves from excessive burden, regulating their level of involvement, and protecting themselves from emotional overload. Numerous studies have confirmed that a strong sense of coherence is linked to better mental well-being and a lower perceived burden or sense of isolation among caregivers. For example, a study by Andrén and Elmståhl [47] reported a strong negative association between these variables. Similar findings have been reported in studies involving caregivers of individuals with Alzheimer’s disease [46], dementia [48,64], Parkinson’s disease [65], and stroke survivors [66].

The results confirm the hypothesis regarding the mediating role of a sense of coherence in the relationship between stress and involvement in caregiving (H4). The moderate indirect effect observed underscores the central role of a sense of coherence as a psychological mechanism that buffers the impact of stress. This suggest that caregivers with a stronger sense of understanding, who are able to find meaning in their experiences and feel confident in their coping abilities, are less likely to respond to caregiving-related stress with excessive involvement. This, in turn, may protect them from becoming overloaded. These findings are consistent with Aaron Antonovsky’s salutogenetic theory [42], which posits that a sense of coherence, as a central resilience resource, enhances an individual’s ability to cope with difficult situations. In this context, stress does not necessarily lead to excessive involvement if the caregiver possesses internal resources that support psychological stability. Caregivers with a strong sense of coherence may be more likely to reinterpret the caregiving situation in a constructive way, which helps them avoid becoming overly involved during periods of heightened stress.

In line with hypothesis H5, the amount of time devoted to caregiving moderated the relationship between sense of coherence and involvement in caregiving. The results of the moderation analysis indicate that the time commitment strengthened the negative association between sense of coherence and caregiving involvement. In other words, the greater the time investment, the stronger the reduction in involvement observed among individuals with a high sense of coherence. For caregivers who devoted less time, this effect was negligible. These findings suggest that, in situations requiring substantial time investment, internal resources—such as a strong sense of coherence—play a key role in regulating emotional involvement. This pattern supports the rationale for adopting a complex interactional model, in which the influence of psychological resources—such as a sense of coherence—is not universal but becomes particularly salient under conditions of elevated stress and overload. These findings are consistent with the theoretical assumptions of the transactional stress model [35], which emphasises that crucial role of personality and cognitive factors in situations that demand substantial coping resources. It can be assumed that caregivers with a strong sense of coherence who devote more time to caregiving are more effective at regulating their level of involvement, which, in turn, helps them avoid overload. These findings highlight the need for targeted support for caregivers providing intensive care, as well as the potential value of interventions aimed at strengthening a sense of coherence. Numerous studies have confirmed that, as the amount of time devoted to caregiving increases, so too does the level of involvement—reflected in a greater number of caregiving activities—which ultimately contributes to a heightened sense of burden [67,68,69].

Moderated mediation was also confirmed (H6), indicating that the indirect effect of stress on involvement in caregiving, mediated by a sense of coherence was stronger among caregivers who devoted more time their caregiving role. This finding provides important evidence of the complex psychological dynamics involved in caregiving and underscores the critical role of contextual factors—particularly the intensity of time commitment—in shaping the relationship between stress, sense of coherence, and caregiving behaviours. According to Aaron Antonovsky’s concept [42], sense of coherence functions as a key meta-resource, particularly in situations involving a high level of burden. Under conditions of intensive time commitment, a sense of coherence becomes increasingly important as a mechanism regulating how stress influences the level of involvement. Caregivers who spend a substantial amount of time providing care are particularly dependent on their resilience resources. The caregiver’s ability to find meaning in their experiences, understand the situation, and maintain confidence in their coping abilities determines how stress is translated into caregiving behaviours. Previous research has highlighted that the amount of time devoted to caregiving each day is one of the key factors influencing the sense of burden experienced by caregivers [14,15,16,17,23,24,26].

The results of our analyses (H7) show that caregivers’ education was significantly associated with sense of coherence and the level of stress they experienced; however, no significant association was found between education and the level of caregiver involvement. The positive relationship between education and a sense of coherence underscores the importance of cognitive resources and competencies in shaping beliefs about the predictability, coherence, and meaningfulness of the surrounding world, as well as one’s own ability to cope with challenges. This finding aligns with Antonovsky’s concept [42], which emphasises the crucial role of life experiences in the development of a sense of coherence. In this context, a higher level of education may facilitate the development of meaning, understanding, and confidence in one’s coping abilities, as well as enhance the ability to comprehend and interpret caregiving experiences. Further analysis revealed that the caregiver’s level of education was negatively associated with the level of stress they experienced. Education may, therefore, function as a protective resource in challenging situations. Greater cognitive competencies, along with planning, problem-solving, and time-management skills, may enable caregivers to cope with stress more effectively. Numerous studies have identified a lower level of education as a significant risk factor associated with an increased sense of burden [70,71]. However, this relationship was not confirmed in the present study. Among the variables analysed, only the stage of illness emerged as a significant predictor of caregiver involvement, supporting the assumption that increasing patient needs expand both the scope and intensity of caregiving duties. These findings are consistent with previous research indicating that caring for individuals in advanced stages of illness leads to a greater caregiver burden [67,68,69], heightened anxiety concerning the care recipient’s condition [72], and an increasing sense of loss freedom and independence among caregivers [73].

## 5. Conclusions

The results of the study provide empirical support for the proposed theoretical model, indicating that the influence of stress on caregiving involvement among individuals caring for people with Alzheimer’s disease is indirectly mediated by a reduced sense of coherence. This relationship was moderated by the amount of time devoted to caregiving—the longer the time commitment, the stronger the indirect effect of stress on involvement. This suggests that caregivers who spend more time providing care are at a greater risk of experiencing an increased sense of burden, particularly if they exhibit a low sense of coherence.

The relationships identified in this study have important practical implications. They underscore the need to strengthen a sense of coherence as a psychological resource that serves a protective role against the negative effects of stress. At the same time, it is essential to consider the context of time-related burden when planning support, particularly for caregivers who are intensively involved in caregiving and, therefore, more vulnerable to mechanisms leading to excessive involvement. The findings highlight the importance of addressing both individual psychological resources and the objective conditions of caregiving. The proposed model may serve as a foundation for developing risk assessment tools and designing interventions aimed at supporting the mental well-being of those caring for individuals with Alzheimer’s disease. The appropriate psychoeducation for caregivers can play an important role in strengthening their sense of coherence. This may involve explaining the mechanism of the disease and the symptoms observed in the person with dementia, learning effective communication skills and ways of responding to challenging situations, and engaging in relaxation technique or mindfulness practice. Participation in support groups can also be beneficial, as it enables caregivers to share their experiences and identify their personal strengths. Integrated resilience training programmes may further contribute to an increase in sense of coherence and a reduction in stress levels.

### Limitations

The present study has several important limitations that should be considered when interpreting the findings. The relatively small sample size (*n* = 100), the uneven gender distribution with a predominance of female caregivers (*n* = 78) (which reflects the caregiver structure in Poland), and the predominance of care recipients in stage II of the disease (*n* = 64) may limit the statistical power of the analyses and the generalisability of the findings. The cross-sectional design employed in the study does not allow for any causal inferences regarding the relationships between the variables. Voluntary participation may have introduced selection bias, potentially leading to an overrepresentation of caregivers who are more engaged in their caregiving role or who possess greater coping resources. Additionally, recruitment conducted in clinical settings may have influenced the characteristics of the sample, excluding caregivers of undiagnosed individuals or those outside the healthcare system. Despite these limitations, the findings provide a significant contribution to understanding the psychological processes underpinning caregiver involvement.

The findings of this study should be interpreted within the context of Polish cultural conditions, where, due to the lack of professional institutional facilities, the burden of care falls primarily on the family on the person with dementia, particularly on women [74]. Limited access to institutional support may contribute to higher levels of caregiver burden and stress compared to countries with more developed formal care system. These factors may limit the generalizability of the results to caregivers functioning in different cultural contexts.

## Figures and Tables

**Table 1:** Descriptive statistics (*n* = 100).

Variable	Minimum	Maximum	Mean	Standard Deviation	Skewness	Kurtosis	VIF	Alpha-Cronbach’s Coefficient
Stress	25	80	53.72	14.48	−0.04	−0.86	1.52	0.84
Sense of coherence	96	188	139.29	20.41	−0.11	−0.29	1.53	0.91
Time devoted to caregiving	2	4	3.44	0.72	−0.90	−0.55	1.03	
Involvement in caregiving	0.39	3.16	1.78	0.63	0.22	−0.53		0.92

VIF—Variance inflation factor.

**Table 2:** Values of r-Pearson correlation coefficients between research variables (*n* = 100).

	1	2	3	4	5	6	7
1. Stress							
2. Sense of coherence	−0.58 **						
3. Involvement in caregiving	0.21 *	−0.27 **					
4. Time devoted to caregiving	0.13	−0.17	0.23 *				
5. Age	−0.01	0.10	−0.13	0.05			
6. Education	−0.29 **	0.25 **	0.01	−0.12	0.09		
7. Duration of caregiving	0.09	0.03	0.24 *	0.03	−0.10	−0.13	
8. Stage of illness	0.09	−0.06	0.59 **	0.09	−0.12	0.03	0.49 **

* *p* < 0.05. ** *p* < 0.01.

**Table 3:** Results of direct effects for a 95% confidence interval.

Pathway	Value of Effect	*p*	LLCI	ULCI
Stress—sense of coherence	−0.774	0.000	−1.013	−0.535
Sense of coherence—involvement in caregiving	−0.006	0.032	−0.012	−0.001
Stress—involvement in caregiving	0.001	0.751	−0.007	0.009
Time devoted to caregiving—involvement in caregiving	0.158	0.025	0.019	0.297
Age—involvement in caregiving	−0.005	0.222	−0.012	0.002
Education—involvement in caregiving	0.123	0.146	−0.044	0.291
Time devoted to caregiving—involvement in caregiving	−0.001	0.916	−0.028	0.025
Stage of illness—involvement in caregiving	0.534	0.000	0.341	0.728

LLCI = 95% confidence interval (low); ULCI = 95% confidence interval (high).

**Table 4:** Results of moderation analyses.

	Moderating Variable	Interaction Effect	Coefficient	SE	t	*p*	LLCI	ULCI
(Outcome: involvement in caregiving)	Time devoted to caregiving	Sense of coherence x Time devoted to caregiving	−0.008	0.003	−2.38	0.019	−0.0149	−0.0014

Note. LLCI = 95% confidence interval (low); ULCI = 95% confidence interval (high).

**Table 5:** Moderating effects of stress on involvement in caregiving for different values of time devoted to caregiving as the moderator.

Time Devoted To Caregiving	Effect	SE	LLCI	ULCI
−0.79 (−1 *SD*)	0.001	0.003	−0.0071	0.0061
0.00 (*M*)	0.005	0.002	0.0004	0.0101
0.56 (+1 *SD*)	0.009	0.003	0.0030	0.0148

Note. LLCI = 95% confidence interval (low); ULCI = 95% confidence interval (high).

## Data Availability

All data have been made publicly available at “https://osf.io/3c92p/?view_only=b1dedff5f0a34ee7a07684703bf5218c (accessed on 23 March 2025)”.
